# Mutation profile and chromosomal abnormality in adenomyosis

**DOI:** 10.1530/REP-25-0132

**Published:** 2025-07-18

**Authors:** Kazuaki Suda, Kotaro Takahashi, Ryo Tamura, Kyota Saito, Manako Yamaguchi, Nozomi Yachida, Sosuke Adachi, Hiroaki Kase, Shujiro Okuda, Kosuke Yoshihara, Hirofumi Nakaoka

**Affiliations:** ^1^ Department of Obstetrics and Gynecology, Niigata University Graduate School of Medical and Dental Sciences, Niigata, Japan; ^2^ Department of Cancer Genome Research, Sasaki Institute, Sasaki Foundation, Chiyoda-ku, Tokyo, Japan; ^3^ Department of Pediatrics, Child Health Research Center, University of Virginia School of Medicine, Charlottesville, Virginia, USA; ^4^ Department of Obstetrics and Gynecology, Nagaoka Chuo General Hospital, Nagaoka, Japan; ^5^ Medical AI Center, Niigata University School of Medicine, Niigata, Japan; ^6^ Department of Biomedical Data Science, Kagoshima University Graduate School of Medical and Dental Sciences, Kagoshima, Japan

**Keywords:** adenomyosis, somatic mutation, chromosome 1q, uterine endometrium, next-generation sequencing

## Abstract

**In brief:**

By tissue-selective next-generation sequencing, we showed that adenomyotic epithelium harbored genomic alterations thought to be relevant to the development and progression of adenomyosis, including somatic mutations in several cancer-associated genes with high mutant allele frequencies and the gain of chromosome 1q. Clonal relationships among multiple adenomyotic lesions and the normal uterine endometrium delineate the oligoclonal origin of adenomyosis and the spatial expansion of mutant clones.

**Abstract:**

To identify the distinctive features of mutation profiles in adenomyosis compared to the coexisting normal endometrium, multi-regional sampling was performed to collect samples of adenomyotic epithelium (*n* = 41), adenomyotic stroma (*n* = 12), and uterine endometrial epithelium (*n* = 53) from 21 patients with adenomyosis. To enhance the purity in this genomic study, laser microdissection was used to isolate all the samples. Target-gene sequencing and whole-exome sequencing were performed to identify somatic mutations in cancer-associated genes and the pattern of cellular expansion in adenomyosis and clonality between adenomyosis and uterine endometrium. In adenomyotic epithelium, we identified somatic mutations in cancer-associated genes such as *KRAS* (34.1%), *PIK3CA* (12.2%), *ARID1A* (12.1%), and *FBXW7* (9.8%) with high mutant allele frequency. In uterine endometrial epithelium, frequently mutated genes included *KRAS* (47.2%), *PIK3CA* (37.8%), and *ARHGAP35* (28.3%). Whole-exome sequencing revealed clonal relationships among adenomyotic lesions, and between adenomyosis and uterine endometrium. The analysis of somatic copy number alterations (SCNAs) showed recurring gain of chromosome 1q in the adenomyotic epithelium but not in the uterine endometrial endometrium. Mutational signature analysis for SNVs revealed that similar mutational processes were shared in adenomyosis and uterine endometrium. In this study, we identified multiple cancer-associated gene mutations and SCNAs relevant to the development of adenomyosis, and also clonal relationships among multiple adenomyotic lesions and normal uterine endometrium.

## Introduction

Adenomyosis, one of the most common gynecological diseases, is characterized by endometrial epithelial and stromal cells within the myometrium. This disease severely diminishes the quality of life by causing heavy menstrual bleeding, dysmenorrhea, dyspareunia, chronic pelvic pain, and infertility (Razavi *et al.* 2019, Kho *et al.* 2021). While hysterectomy offers a definitive cure, drug therapies and uterine-sparing procedures are often preferred, depending on symptom severity and patient’s fertility goals. Most pharmacological treatments focus on modulating estrogen and progesterone levels to reduce endometrial proliferation and inflammation (Kho *et al.* 2021). However, adenomyosis often involves excessive estrogen formation and progesterone resistance. Consequently, effective treatment options for adenomyosis are still lacking (Mehasseb *et al.* 2011, Abbott 2017, Kho *et al.* 2021). Although several hypotheses have been proposed regarding the pathogenesis of adenomyosis – such as the invasion and infiltration of endometrial tissue, transformation from Müllerian duct remnants, and infiltration of endometriosis from the uterine serosal surface – the exact mechanisms remain unclear (Garcia-Solares *et al.* 2018). Understanding the pathogenesis of adenomyosis is clinically crucial to prevent its development and manage its symptoms in women of reproductive age.

Genetic analyses have indicated the involvement of mutations in oncogenes, such as *KRAS* and *PIK3CA*, and abnormal gene expression related to excessive estrogen formation and progesterone resistance in adenomyosis (Vannuccini *et al.* 2017, Inoue *et al.* 2019, Bulun *et al.* 2021, Chao *et al.* 2023). Notably, Inoue *et al.* identified somatic *KRAS* mutations in 26 (37.1%) of 70 adenomyosis cases. In addition, *KRAS* mutations were detected in coexisting endometriosis and normal endometrium in several subjects, suggesting that both adenomyosis and endometriosis may originate from normal endometrial tissue. Furthermore, the presence of *KRAS* mutation has been associated with decreased progesterone receptor (PgR) expression, indicating that adenomyotic tissues with *KRAS* mutation may exhibit progesterone resistance (Inoue *et al.* 2019). A significant limitation of previous studies is that most analyzed samples were collected using manual macrodissection rather than more precise methods, such as laser microdissection (LMD). As adenomyotic tissue exhibits heterogeneous distribution within the uterine smooth muscle tissue, manual macrodissection can lead to lower purity of adenomyotic epithelial cells, which potentially reduces the accuracy of detecting somatic mutations and somatic copy number alterations (SCNAs) and evaluating clonal relationships among adenomyotic lesions and normal endometrium. Thus, selective tissue sampling is necessary to ensure precision in the genetic analysis of adenomyosis. Indeed, our previous research demonstrated that comprehensive genetic analyses of endometriosis, normal endometrium, and endometriosis-associated ovarian cancer using LMD were effective in identifying their genetic characteristics and clonal evolution (Suda *et al.* 2018, Suda *et al.* 2020, Yamaguchi *et al.* 2022).

Recently, we provided further evidence supporting the hypothesis that normal endometrium is the origin of adenomyosis by demonstrating, through three-dimensional imaging, the direct infiltration of endometrial epithelium into the myometrium in a patient with adenomyosis (Yamaguchi *et al.* 2021). In this study, we performed target-gene sequencing (TS) and whole-exome sequencing (WES) on 41 epithelial and 12 stromal samples of adenomyosis, and 53 uterine endometrial glands from 21 patients, all of which were isolated using LMD. The genomic analysis, which was based on the selective sampling of epithelial and stromal compartments in adenomyosis and uterine endometrium, facilitated exploring the detailed profiles of cancer-associated gene mutations, clonal relationships, chromosomal abnormalities, and mutational signatures. We believe that the findings of this study will provide new insights into the genomic landscape of uterine adenomyosis and contribute toward elucidating its pathogenesis.

## Materials and methods

### Human samples and study design

This study was approved by the institutional ethics review boards of Niigata University, Nagaoka Chuo General Hospital, and Sasaki Institute. We recruited study participants at the Niigata University Medical and Dental Hospital and the Nagaoka Chuo General Hospital between August 2015 and August 2018. All subjects provided written informed consent for sample collection, data analysis, and publication of their clinical information.

We conducted multi-regional sampling on 21 subjects with adenomyosis aged 36–56, who underwent hysterectomy, and collected 41 adenomyotic and 53 uterine endometrial epithelial samples. In addition, paired stromal samples were obtained from 12 of 21 subjects with adenomyosis. Peripheral blood samples were also collected from each subject to serve as a reference for somatic mutation analysis. Detailed clinicopathological information for the subjects is provided in the Supplementary Table 1 (see the section on Supplementary materials given at the end of the article).

Our study design consisted of two steps. First, we performed TS on all samples to detect representative somatic mutations in cancer-associated genes within adenomyosis and uterine endometrium. Second, we conducted WES on selected samples to investigate clonal relationships, copy number alterations, and mutational signatures. Sample selection for WES was based on TS results by focusing on cases where somatic mutations were shared among different adenomyotic samples or between adenomyotic and uterine endometrial samples from the same subject, and where sufficient DNA was available.

### Laser microdissection (LMD)

**Figure 1:**
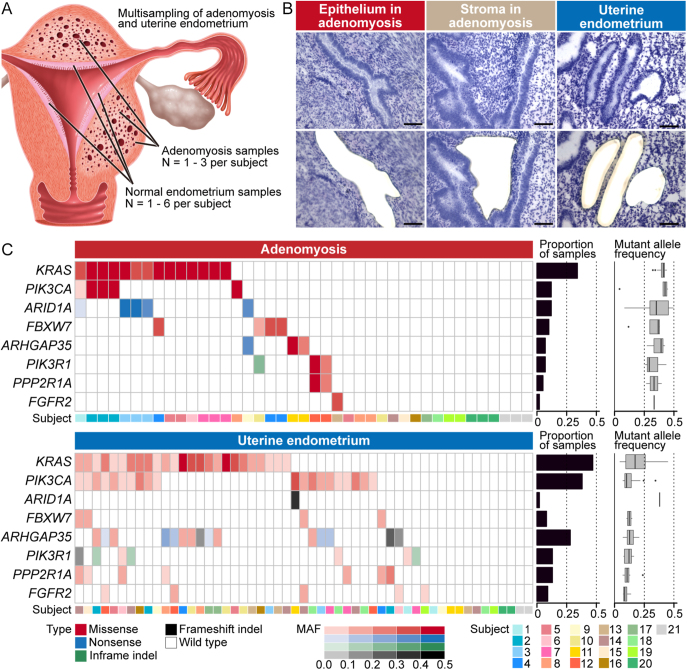
Tissue sampling and mutation landscapes of adenomyosis and uterine endometrium based on target-gene sequencing. (A) Schematic overview of tissue sampling from subjects with adenomyosis in this study. (B) Representative images showing tissue-selective sampling by laser microdissection for epithelium in adenomyosis, stroma in adenomyosis, and uterine endometrium. The upper and lower panels display histological images before and after laser microdissection, respectively. Scale bars represent 100 μm. (C) Mutation landscapes of adenomyosis and uterine endometrium. Heatmaps show the distribution of recurrent mutations of eight representative cancer-associated genes. The color and its density indicate type of mutation and MAF, respectively. Bar graphs depict the proportion of samples harboring mutations, stratified by genes. Box-whisker plots present variations in mutant allele frequencies of somatic mutations, also stratified by genes.

All subjects were pathologically diagnosed with adenomyosis with no uterine malignancy by a proficient pathologist. Before LMD, we histologically examined adenomyotic and uterine endometrial samples using frozen sections that were fixed with 4% paraformaldehyde and stained with hematoxylin–eosin. We performed LMD as described in our previous studies (Suda *et al.* 2018, Suda *et al.* 2019). Briefly, 10 μm-thick serial frozen sections (number of sections ranged 2–20) were mounted on membrane slides (Leica Microsystems, Germany), fixed with 100% ethanol, rapidly stained with toluidine blue, and washed by nuclease-free water on ice. Using the LMD7 LMD microscope (Leica Microsystems), we isolated 41 adenomyotic epithelial, 12 paired stromal, and 53 uterine endometrial epithelial samples (Fig. 1).

### DNA extraction

We extracted DNA from the microdissected samples using the QIAamp DNA Micro Kit (Qiagen, Germany). In addition, DNA from peripheral blood samples was extracted using the QIAamp DNA Blood Maxi Kit (Qiagen).

### Target-gene sequencing (TS)

TS for 112 genes (all symbols and names are listed in the Supplementary Table 2) was performed as described in our previous studies (Ahmadloo *et al.* 2017, Suda *et al.* 2018, Yamaguchi *et al.* 2022), with some modifications. These 112 genes were selected based on the following criteria: WES data for ovarian endometriosis and normal uterine endometrium (Suda *et al.* 2018), genomic analysis for endometriosis-related ovarian cancer (Jones *et al.* 2010) and endometrial cancer (Lawrence *et al.* 2014), and genes involved in DNA repair pathways (The Cancer Genome Atlas Research Network 2011, Norquist *et al.* 2016). DNA samples were fragmented using the KAPA Frag Kit (KAPA Biosystems, USA). We constructed sequencing libraries using the NEBNext Ultra II DNA Library Prep Kit for Illumina (New England Biolabs, USA). Libraries of up to 96 samples were pooled in equimolar amounts and hybridized to probes of a SeqCap EZ Prime Choice System (Roche Diagnostics, Switzerland) in a single enrichment reaction. The DNA probe set was selected using NimbleDesign (Version 3.8) (http://design.nimblegen.com). The quantity and size distribution of the libraries were assessed using a Qubit 2.0 Fluorometer (Thermo Fisher Scientific, USA) and a Bioanalyzer 2100 (Agilent Technologies, USA), respectively. The libraries were then sequenced via the Illumina HiSeq 2500 platform using the 2 × 100-bp paired-end module (Illumina, USA).

### Whole-exome sequencing (WES)

We performed WES following the methods described in our previous study (Suda *et al.* 2020, Tamura *et al.* 2023, Takahashi *et al.* 2024). DNA samples were first repaired with the NEBNext FFPE DNA Repair Mix (New England Biolabs) and then fragmented using the NEBNext dsDNA Fragmentase (New England Biolabs). Sequencing libraries were constructed using the NEBNext Ultra II DNA Library Prep Kit for Illumina (New England Biolabs). Target-gene enrichment was conducted with the IDT xGen Exome Research Panel v2 (Integrated DNA Technologies, USA). The libraries were sequenced via an Illumina NovaSeq 6000 platform with the 2 × 150 bp paired-end module (Illumina).

### Data pre-processing

As a part of quality control, we trimmed the Illumina adapter sequences using TrimGalore (Version 0.6.3) (https://www.bioinformatics.babraham.ac.uk/projects/trim_galore/). Low-quality sequences were either excluded or trimmed using Trimmomatic (version 0.39) (Bolger *et al.* 2014). The filtered sequence reads were then aligned to the human reference genome (GRCh38), which contains sequence decoys and virus sequences generated by the Genomic Data Commons of the National Cancer Institute, using BWA-MEM (Version 0.7.17) (Li *et al.* 2009, Li 2013). The resulting sequence alignment map files were sorted and converted to binary alignment map files using SAMtools (version 1.9) (Li *et al.* 2009). PCR duplicates were removed using Picard tools (version 2.20.6) (http://broadinstitute.github.io/picard/), and base quality recalibration was performed using GATK (version 4.1.3.0) (McKenna *et al.* 2010, DePristo *et al.* 2011). We calculated the average depths and coverages of the target regions using SAMtools (Li *et al.* 2009). For handling FASTA, VCF, and BED files, we used BEDOPS (version 2.4.36) (Neph *et al.* 2012) and BEDTools (v2.28.0) (Quinlan & Hall 2010). The average depths and coverages of TS and WES are shown in the Supplementary Tables 3 and 4, respectively.

### Variant detection and mutation annotation

Somatic single-nucleotide variants (SNVs) and short insertions/deletions (Indels) in coding exons and splice sites were identified using Strelka2 (version 2.9.10) (Kim *et al.* 2018). For somatic Indel detection, we used candidate Indel sites suggested by Manta (version 1.6.0) (Chen *et al.* 2016). Variants were considered for further analysis if they had an empirical variant scores of >13.0103 (= −10 × log_10_ 0.05) from Strelka2. To avoid false-positive variant calls, we excluded variants with frequencies ≥0.001 in any of the general populations from the 1000 Genomes Project (Auton *et al.* 2015), the National Heart, Lung, and Blood Institute GO Exome Sequencing Project (Tennessen *et al.* 2012), and the Genome Aggregation Database (Karczewski *et al.* 2020). Functional annotations for the identified variants, including their effects on protein coding and transcription, were implemented using Ensembl VEP (McLaren *et al.* 2016). Curated information regarding cancer-associated genes and their functional roles in cancer development was retrieved from the COSMIC database (Tate *et al.* 2019).

### Evaluation of mutation sharing among samples

To evaluate the extent of shared somatic mutations among samples from different spatial regions using WES data, we compiled mutant allele frequency (MAF) profiles of all the mutation sites for each sample by counting the sequence reads supporting both the reference and mutant alleles using SAMtools mpileup (Li *et al.* 2009). For this analysis, we used reads mapped with high confidence (mapping quality >30). Allele-specific counts were measured using only high-confidence base calls (base quality >20) at the mutation sites. We excluded sites where MAFs in the blood samples exceeded 0.05. Informative mutations were selected based on MAF values of at least 0.15 and 0.10 in adenomyosis and uterine endometrium, respectively.

### Identification of putative clonal populations

We detected clonal populations within adenomyosis and uterine endometrium samples of each subject by clustering somatic mutations (SNVs and Indels) using PyClone (Roth *et al.* 2014). The selection criteria for SNVs and Indels included: i) mutations used for MAF profiles based on WES data, as described above; ii) a sequencing depth greater than or equal to 10 across all samples from a subject; and iii) exclusion of the mutations overlapping with SCNA detected by FACETS. The clustered SNVs and Indels along with their MAFs were then used as input into ClonEvol (Dang *et al.* 2017). We employed the polyclonal seeding model, with the number of bootstrap samples set to 10,000.

### Detection of somatic copy number alterations (SCNAs)

We sought SCNAs using FACETS, as per the information on total sequence read counts and allelic imbalances in adenomyosis or uterine endometrium samples, compared to matched blood samples (Shen & Seshan 2016). Germline polymorphic sites were retrieved from the VCF file generated by the 1000 Genomes Project (Auton *et al.* 2015). The cutoff for an SCNA detection was set at an estimated cellular fraction of 0.3 or higher, with the length of SCNA being one mega base or more.

### Mutational signature analysis

We utilized the somatic SNVs selected by WES analysis for mutational signature analysis in adenomyosis and uterine endometrium. Owing to the small number of SNVs per sample, we aggregated somatic mutations for all adenomyosis or uterine endometrium samples into a single entity. The mutations shared among multiple samples from a single subject were treated as one. The somatic SNVs were classified into 96 mutation classes based on the six possible pyrimidine substitutions (C>A, C>G, C>T, T>A, T>C, and T>G) combined with the flanking 5' and 3' bases. This 96-mutation catalog was then compared to a predefined list of known mutational signatures (Baez-Ortega *et al.* 2019, Maura *et al.* 2019). We did not perform *de novo* signature extraction because of the limited number of somatic mutations. Instead, we used the COSMIC mutational signatures version 3 as a reference set of known mutational signatures (Alexandrov *et al.* 2020) and selected ten single-base substitution (SBS) signatures (SBS1, SBS2, SBS5, SBS10a, SBS10b, SBS13, SBS15, SBS18, SBS21, and SBS44) that were relevant to three gynecologic cancers (cervical cancer, endometrial cancer, and ovarian cancer) (https://cancer.sanger.ac.uk/cosmic/signatures/). A fitting approach was applied using sigfit (Gori & Baez-Ortega 2020). We ran four Markov chains with a total of 50,000 iterations, including a burn-in of 25,000 iterations. The highest posterior density (HPD) interval was estimated for each SBS signature. An SBS signature was considered significantly active if the 90% lower end of the HPD interval for the corresponding SBS signature exceeded the threshold (default value of 0.01).

### Statistical analysis

All statistical analyses were performed using the R program. Comparisons were performed using the Wilcoxon exact rank test (exactRankTests) and Fisher exact test (stats) as appropriate. *P* < 0.05 was considered statistically significant.

## Results

### Cancer-associated gene mutations in adenomyosis and uterine endometrium

We performed TS of 112 genes in 43 adenomyosis and 51 uterine endometrium samples from 21 subjects, aged 36–56 years. Over half of the samples from both adenomyosis and uterine endometrium contained somatic mutations in genes that are commonly mutated in endometrial cancer and endometriosis-associated ovarian cancer (Fig. 1C, Supplementary Table 5). In adenomyosis, the most frequently mutated genes included *KRAS* (34.1%), *PIK3CA* (12.2%), *ARID1A* (12.1%), and *FBXW7* (9.8%). The MAFs of representative cancer-associated genes in adenomyosis exceeded 0.25, with *KRAS* and *PIK3CA* showing averages of 0.42 and 0.43, respectively, indicating their clonal expansion in adenomyotic lesions. No mutations in MAFs exceeded 0.5, suggesting that these mutations did not accompany allelic imbalances. All *KRAS* mutations were located within the hotspot regions (Supplementary Fig. 1). In uterine endometrium, the most recurring mutated genes were *KRAS* (47.2%), *PIK3CA* (37.8%), *ARHGAP35* (28.3%), *PIK3R1* (13.2%), *PPP2R1A* (13.2%), and *FGFR2* (9.4%). The MAFs of key cancer-associated genes in uterine endometrium were below 0.25. Individual uterine endometrial glands are considered monoclonal in origin and harbor distinct somatic mutations (Tanaka *et al.* 2003, Moore *et al.* 2020, Yamaguchi *et al.* 2022). Thus, this result could be explained by the sampling method, in which multiple uterine endometrial glands were collected from each specimen.

**Figure 2:**
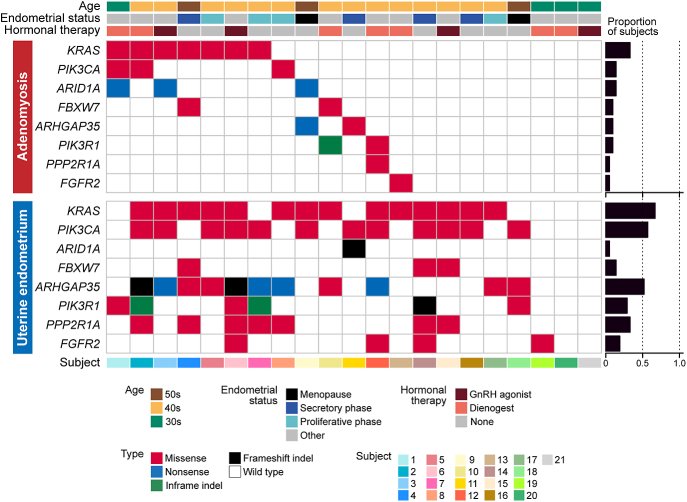
Summary of somatic mutations detected by target-gene sequencing in each subject. The heatmap shows clinical information and the distribution of recurrent somatic mutations of eight cancer-associated genes. In the subject with multiple types of the same mutations, the one with higher MAF was adopted. Bar graphs show the proportion of subjects harboring mutation stratified by genes.

The mutation frequencies per subject, regardless of sampling depths, were as follows: in adenomyosis, *KRAS*: 33.3%, *PIK3CA*: 14.3%, and *ARID1A*: 14.3%, and in uterine endometrium, *KRAS*: 66.7%, *PIK3CA*: 57.1%, *ARHGAP35*: 52.4%, *PPP2R1A*: 33.3%, *PIK3R1*: 28.6%, and *FGFR2*: 19.0% (Fig. 2). A comparison of clinical information between seven subjects with *KRAS* mutation and 14 subjects without *KRAS* mutation in adenomyosis revealed no significant differences in age, coexistence of endometriosis, hormonal therapy (dienogest or GnRH agonist), pregnancy history (gravidity, parity, and cesarean section), or the history of curettage procedure (Supplementary Table 6).

The TS analysis of the stromal samples from 12 patients with adenomyosis detected only five somatic mutations, even when silent mutations were included (Supplementary Table 7). None of these mutations were observed in the adjacent adenomyotic epithelium, and vice versa, suggesting that the stroma and epithelium in adenomyosis originated from distinct cells of origins.

### Shared cancer-associated gene mutations identified by TS

**Figure 3:**
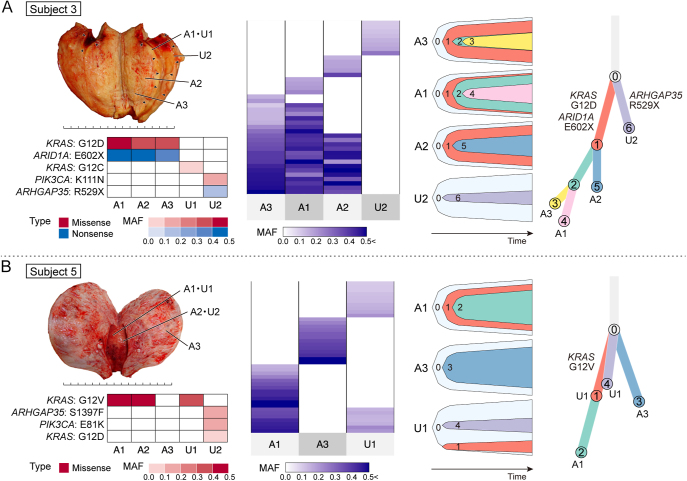
Clonal relationships in representative subjects with adenomyosis. (A) Subject 3 presents a widespread identical clone within adenomyosis. (B) Subject 5 exhibits two distinctive features: independent clones within adenomyosis and a shared clone between adenomyosis and uterine endometrium. In both (A) and (B), the upper left panel shows the macroscopic image of a surgical specimen with sampling sites. Each interval in a scale bar represents 1 cm. The lower left panel displays a heatmap of mutation profiles of representative cancer-associated genes detected by target-gene sequencing. Color and its density reflect type of mutation and MAF), respectively. The middle panel illustrates the shared pattern of somatic mutations detected by WES. Each line represents distinct somatic mutation, and color density indicates MAF. The right panel shows fish plots and a branch-based clonal evolution tree by clustering with PyClone and ordering with ClonEvol, based on WES results. Fish plots depict the process of clonal evolution in each sample. In the clonal evolution tree, mutations in cancer-associated genes were assigned to branches based on mutation clustering by PyClone. Sample identifiers are indicated beside a node if the corresponding clone was observed in the samples when taken. The branch lengths correlate with the number of somatic mutations. A and U denote adenomyosis and uterine endometrium, respectively.

We investigated shared mutations among multiple samples from each subject based on their TS mutation profiles (Fig. 3, Supplementary Fig. 2). In eight subjects (subjects 2, 3, 4, 5, 7, 11, 12, and 20), multiple adenomyotic lesions had the same somatic mutations in cancer-associated genes. *KRAS* mutations were shared in four subjects, while *ARID1A*, *PIK3CA*, *FBXW7*, *ARHGAP35*, *PIK3R1*, *PPP2R1A*, and *TAF1* mutations were each shared in one subject. In addition, three subjects harbored identical *KRAS* mutations shared between adenomyosis and uterine endometrium (subjects 2, 5, and 6), with one subject also sharing a *PIK3CA* mutation between these tissues.

### Clonal relationships in adenomyosis revealed by WES

We hypothesized that a larger number of somatic mutations identified by WES would provide a more detailed understanding of clonal relationships. Thus, we additionally performed WES analyses on samples from subjects (subjects 2, 3, 4, 5, 6, 7, 11, 12, and 20) who had shared mutations on cancer-associated genes across multiple adenomyotic lesions or between adenomyosis and uterine endometrium. Unfortunately, we could not perform WES on several samples because of insufficient DNA. As a consequence, 16 adenomyosis and five uterine endometrium samples from seven subjects (subjects 2, 3, 4, 5, 6, 7, and 20) were subjected to WES. Initially, we examined the patterns of shared mutations detected by WES among samples in each sample. Second, we reconstructed clonal evolution trees using PyClone and ClonEvol to explore clonal relationships among the samples.

**Figure 4:**
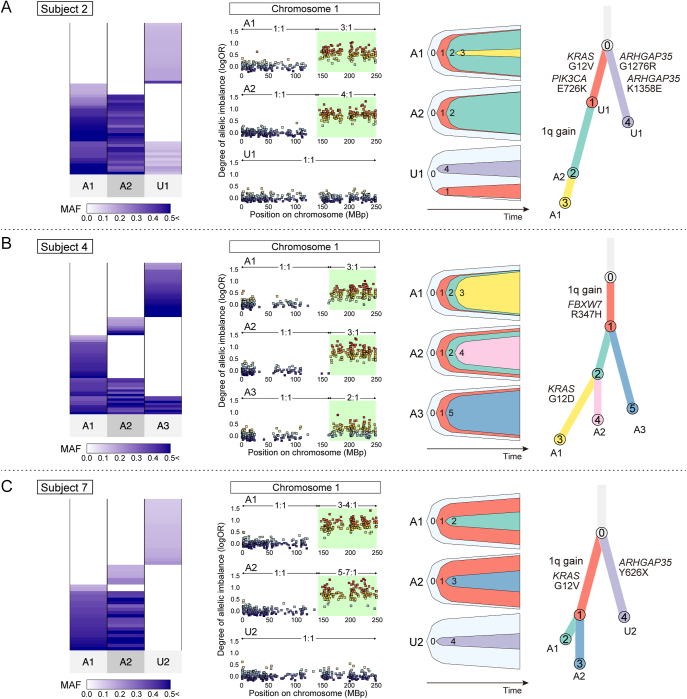
Clonal relationships in subjects with adenomyosis exhibiting chromosome 1 gain. Adenomyosis samples with a gain of chromosome 1 were identified in subjects 2 (A), 4 (B), and 7 (C). The left panel in each subject shows the shared pattern of somatic mutations detected by WES. Color density represents the MAF of each somatic mutation. The middle panel depicts SCNAs on chromosome 1. The log odds ratios were estimated using FACETS. In each case, the adenomyosis sample exhibited a gain of the long arm of chromosome 1. The right panel displays fish plots and a branch-based clonal evolution tree, as described. The events of chromosome 1 gain were manually assigned according to the SCNA analysis. A and U indicate adenomyosis and uterine endometrium, respectively.

Owing to the increased number of somatic mutations detected via WES, the patterns of shared mutations became much clearer compared to those with TS (Fig. 3, Supplementary Fig. 3, Supplementary Tables 8 and 9). In subjects 2, 3, 4, 7 and 20 (Figs 3A, 4 and Supplementary Fig. 3), a significant number of mutations were shared among multiple adenomyotic lesions. In addition, each sample accumulated unique mutations and showed substantial heterogeneity among multiple adenomyotic lesions. The clonal relationship analysis revealed that cluster 1 represented a common ancestral clone (Figs 3A, 4 and Supplementary Fig. 3). This cluster contained mutations in representative cancer-associated genes such as *KRAS*, *ARID1A*, *PIK3CA*, and *FBXW7*. Furthermore, several subclones emerged from this ancestral clone by acquiring unique somatic mutations. These findings suggest that the development of adenomyosis was driven by an ancestral clone carrying mutations in cancer-associated genes, with its subsequent subclones expanding the affected myometrial area through additional mutations. In particular, the ancestral clone (cluster 1) of subject 4 gained the long arm of chromosome 1 (chr1q) and a missense mutation on *FBXW7*. It subsequently acquired a *KRAS* p.G12D mutation and gave rise to a subclone (cluster 3) that was unique to one of the three adenomyosis samples (A1) (Fig. 4B). This suggests that the gain of chr1q and *FBXW7* mutation were initial driving events in disease development, with the *KRAS* mutation acting as a subclonal driver colonizing the sampling area (A1). Conversely, mutation profiles were entirely different between several pairs of adenomyosis sampling sites in subjects 5 and 20 (Fig. 3B, Supplementary Fig. 3). This indicates that multiple mutant clones arose independently to form distinct adenomyotic foci.

In subjects 2 and 5 (Fig. 3B and 4A), adenomyosis and uterine endometrium shared a substantial number of mutations. Clonal relationship analysis in these subjects demonstrated that an ancestral clone (cluster 1) had clonally expanded in adenomyosis but constituted only a small fraction of cell population in the uterine endometrium. This disparity could be attributed to the uterine endometrium samples containing multiple endometrial glands. Thus, these results support evidence that the ancestral clone (cluster 1) in the uterine endometrium was the origin of adenomyosis. However, adenomyosis and uterine endometrium of subject 6 shared only one mutation (*KRAS* p.G12V) and none other (Supplementary Fig. 3). This observation suggests two plausible explanations: i) adenomyosis originated from epithelial cells contained in the sampled uterine endometrium, where the *KRAS* mutation initially occurred, followed by divergent clonal evolution in adenomyosis and uterine endometrium as additional mutations independently accumulated, or ii) adenomyosis did not originate from epithelial cells contained in the sampled uterine endometrium, and the same *KRAS* mutation independently occurred in both tissues. This finding implies that the presence of a shared hotspot mutation on a cancer-associated gene is insufficient to precisely infer clonal relationship. The clonal evolution analysis suggests a scenario in which one of the uterine endometrial glands infiltrated the uterine myometrium and led to the development of adenomyosis.

### SCNAs in adenomyosis

SCNA analysis using FACETS revealed that three subjects with adenomyosis (subjects 2, 4, and 7) exhibited a gain of chr1q in adenomyosis (Fig. 4, Supplementary Table 10). Moreover, this chr1q gain was consistently shared across multiple adenomyotic lesions in these subjects. In contrast, paired uterine endometrial samples did not harbor the chr1q gain, even though several mutations were shared between adenomyotic and uterine endometrial samples in subject 2. Moreover, the chr1q gain was the only SCNA that was shared among multiple adenomyotic lesions. These findings suggest that the gain of chr1q may promote the development of adenomyosis. Unlike our previous studies on endometriosis and endometrial glands (Suda *et al.* 2018, Yamaguchi *et al.* 2022), we could not detect any loss of heterozygosity (LOH) in adenomyosis. In addition, no mutation in cancer-associated genes was found in the SCNA regions, including the gain of chr1q, in any of the adenomyosis samples. However, we detected a nonsense mutation in *PTEN* in the LOH region of chromosome 10 in the uterine endometrium of subject 2. The MAF of *PTEN* was 0.40, which was higher than the average of MAFs (0.15) for other mutations in this subject’s uterine endometrium, suggesting the bi-allelic loss of *PTEN*.

### Mutational signature analysis in adenomyosis

**Figure 5:**
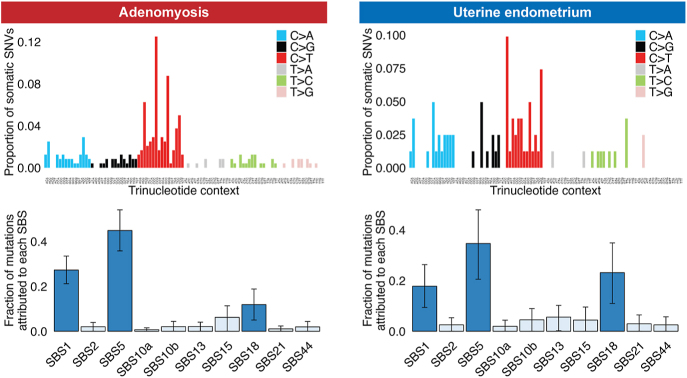
Mutational spectrum and fraction of mutations attributed to each SBS. The upper panels represent the mutation spectrum in adenomyosis and uterine endometrium. Each of the six possible point mutations is further subdivided into 16 subclasses based on the neighboring nucleotides. The lower panels indicate the fraction of mutations attributed to each SBS in adenomyosis and uterine endometrium. The bar chart shows the results of Bayesian inference using sigfit to determine the contribution of COSMIC mutational signatures to somatic single-nucleotide variants. The data are presented as the estimated contributions of significant mutational signatures, along with the lower and upper limits of the 90% HPD interval. Significantly involved signatures are highlighted in dark blue. Source data are provided in the Supplementary Table 12.

To explore the mutational processes underlying adenomyosis, we conducted a mutational signature analysis for SNVs in both adenomyosis and uterine endometrium using WES data (Fig. 5, Supplementary Tables 11 and 12). Three mutational signatures (SBS1, SBS5, and SBS18) were significantly overrepresented in both adenomyotic and uterine endometrial tissues. SBS1 is a clock-like mutation signature characterized by C>T transitions at CpG motifs, indicating the deamination of methyl-cytosines. SBS5 is another clock-like mutation signature with unknown etiology. SBS18, represented by C>A transversions, has been attributed to DNA damage induced by reactive oxygen species. These findings suggest that similar mutational processes are at play in adenomyosis and uterine endometrium.

## Discussion

In this study, we enhanced the purity of genomic analysis by isolating adenomyotic epithelial cell samples using LMD. This allowed us to accurately determine the MAFs of somatic mutations and identify clonal proliferation of adenomyotic epithelium with cancer-associated gene mutations as represented by *KRAS* and *PIK3CA* mutations using TS. In addition, uterine endometrium samples displayed somatic mutations with lower MAFs, likely reflecting the sampling method used in this study, where multiple endometrial glands with distinct somatic mutations were collected using LMD. Subsequent WES of multi-regional samples enabled us to explore the monoclonal and polyclonal nature of adenomyosis, furthering our understanding of the evolution and spatial distribution of mutant clones. Moreover, SCNA analysis based on WES data revealed that arm-level amplification on chr1q occurred in the adenomyotic epithelium. Finally, we examined the mutational signatures that were active in adenomyotic epithelium.

*KRAS* mutations have been reported to be predominant in adenomyosis, but mutations in other cancer-associated genes, such as *PIK3CA*, were rare (Inoue *et al.* 2019). Our findings, however, showed that mutations in multiple cancer-associated genes were involved in adenomyosis. The frequency of *KRAS* mutations per sample was 34%, which is generally consistent with the previous report (Inoue *et al.* 2019), verifying *KRAS* mutation as a major driver of adenomyosis. The *PIK3CA* mutation frequency in adenomyosis in this study was 12%, which was higher than that in the previous adenomyosis study (Inoue *et al.* 2019) but lower than that in the normal endometrium (Bulun *et al.* 2021). This implies that *PIK3CA* mutations may not play an important role in the development of adenomyosis. *ARID1A* mutations, which are rarely observed in normal endometrium, were identified in 12% of adenomyosis in this study, as reported in another recent study (Chao *et al.* 2023). Combined with our previous study, which showed a frequency of 10% of *ARID1A* mutations in ovarian endometriosis (Suda *et al.* 2018), *ARID1A* mutations were also assumed to contribute to the progression of ectopic endometrium. Although mutations in *FBXW7*, *ARHGAP35*, *PIK3R1*, and *PPP2R1A* were also detected in adenomyosis, their frequencies were not higher than those in the normal endometrium. Therefore, these mutations may be inherited from the normal endometrium and may not largely contribute to the development of adenomyosis. In line with the study by another group (Moore *et al.* 2020), our previous two independent genomic researches on normal endometrium revealed that *PIK3CA* was the most frequently mutated gene (Suda *et al.* 2018, Yamaguchi *et al.* 2022). However, in this study, we focused on the genomic analysis of normal endometrium limited to the cases of adenomyosis, *KRAS* mutations tended to be more frequent than *PIK3CA* mutations. This finding suggests that *KRAS* mutation in normal endometrium may be a key factor in the pathogenesis of adenomyosis.

We examined the genetic relationship between the epithelium and adjacent stroma in adenomyosis. Similar to findings in endometriosis and normal endometrium (Suda *et al.* 2019), we observed no clonality (sharing mutations) between epithelium and stroma in adenomyosis. In contrast, recent mouse model studies suggest that mesenchymal–epithelial transition plays a significant role in the pathogenesis of adenomyosis (Kay *et al.* 2020, Yoo *et al.* 2020). Therefore, further investigation is needed to explore the relationship between epithelium and stroma at the gene expression level.

A novel finding in our study was the detection of chr1q amplification in adenomyosis samples in three out of seven cases where WES was performed. No prior reports have linked adenomyosis to chr1q amplification. Chr1q amplification is considered a poor prognostic factor in multiple myeloma, and frequently occurs in other cancer types (Girish *et al.* 2023). Chr1q amplification has been detected in 30–40% of endometrial cancers (Cancer Genome Atlas Research Network *et al.* 2013, Berger *et al.* 2018), and has recently been identified as a risk factor in early low-grade endometrioid carcinoma (Safdar *et al.* 2022). Moreover, chr1q amplification is associated with poor prognosis in endometrial carcinomas lacking a specific molecular profile (Momeni-Boroujeni *et al.* 2022). Further studies are required to delineate the mechanisms by which chr1q amplification is involved in the development of adenomyosis.

Using multi-regional sampling, we assessed whether somatic mutations were shared among multiple adenomyotic lesions. Remarkably, we found several cases with entirely different clones depending on the sampling site, indicating an oligoclonal origin of adenomyosis. This implies that certain characteristics of the myometrium might predispose it to the invasion of diverse endometrial cells. Compared with the finding that endometriotic epithelium represented a higher clonality, as shown in our previous study, the oligoclonality in adenomyosis was thought to be distinctive from the nature of ovarian endometriosis. We interpreted one of the important differences in onsets of these two types of ectopic endometrium, ovarian endometriosis and adenomyosis, as their monoclonal and oligoclonal nature, respectively. On the other hand, most adenomyosis cases where mutations were shared among multiple lesions had either a *KRAS* mutation or chr1q gain as the ancestral clone. These genomic alterations could drive the spread of adenomyotic tissue throughout the uterine smooth muscle. Chr1q gain is a genomic alteration included in this study that was not previously observed in normal endometrial epithelium and appears to have been uniquely acquired by adenomyotic tissue.

This study has some limitations. First, LMD cannot be performed on areas where the intima and adenomyosis are directly connected. Three-dimensional imaging analysis revealed the intimal gland duct entering the muscle layer (Yamaguchi *et al.* 2021). If we could directly perform LMD in this area, it would provide a clearer understanding of the relationship between normal endometrium and adenomyosis. Second, LMD for normal endometrial epithelium was conducted in only limited areas of the entire endometrium in this study, which limited our ability to fully characterize the genomes of the normal endometrium in patients with adenomyosis. For example, cases with adenomyosis could harbor *KRAS* mutations in their normal endometrial glands more frequently than those without adenomyosis. To address this, comprehensive genomic analysis of entire normal endometrial glands in each case is needed. Third, the sample size was small. WES was performed on only a subset of cases that shared representative cancer-related genes between samples, which could potentially introduce bias. Therefore, confirming the frequency of 1q amplification is imperative by increasing the number of cases studied. It was also possible that the small sample size prevented us from identifying differences in clinical backgrounds between patients with and without *KRAS* mutations. Finally, genomic and epigenetic statuses of hormone receptors were not investigated.

In conclusion, our genomic study based on selective tissue sampling identified multiple cancer-associated gene mutations and SCNAs relevant to the development of adenomyosis. It also clarified clonal relationships among multiple adenomyotic lesions and normal uterine endometrium. Thus, the findings of this study provide new insights into the mechanisms involved in the pathogenesis of adenomyosis.

## Supplementary materials





## Declaration of interest

The authors declare that there is no conflict of interest that could be perceived as prejudicing the impartiality of the work reported.

## Funding

This work was supported in part by the Japan Society for the Promotion of Science (JSPS) KAKENHI under grant numbers 19K09822, 19K18633, 21K16810, 22K09635, 23H02756, 23K27447, and 24K12597, the Japan Science and Technology Agency (JST) under grant number JPMJFR2218, and the Japan Agency for Medical Research and Development (AMED) categorized as the Project for Whole Implementation to Support and Ensure the female life (WISE) under grant number 22583077.

## Author contribution statement

KS, KY, and HN helped with conceptualization. Methodology was given by KS, KT and HN. KS, KT and HN helped in investigation. Writing of the original draft was done by KS, KT, RT and HN. Writing of the review and editing was done by HN. Funding acquisition was done by KS, SA, KY and HN. KS, RT, K Saito, MY, NY, HK and SO helped with resources. Supervision was done by KY and HN.

## Data availability

The raw sequencing data underlying this article will be shared on reasonable request to the corresponding author.
