# Supplementary material for: Mutation profile and chromosomal abnormality in adenomyosis
Source: Reproduction. 2025 Jul 18;170(2):e250132. doi: 10.1530/REP-25-0132 (PMC12278445; doi:10.1530/REP-25-0132)
Supplement: Supplementary file 2 [file supplementary_figures_1-3.pdf]

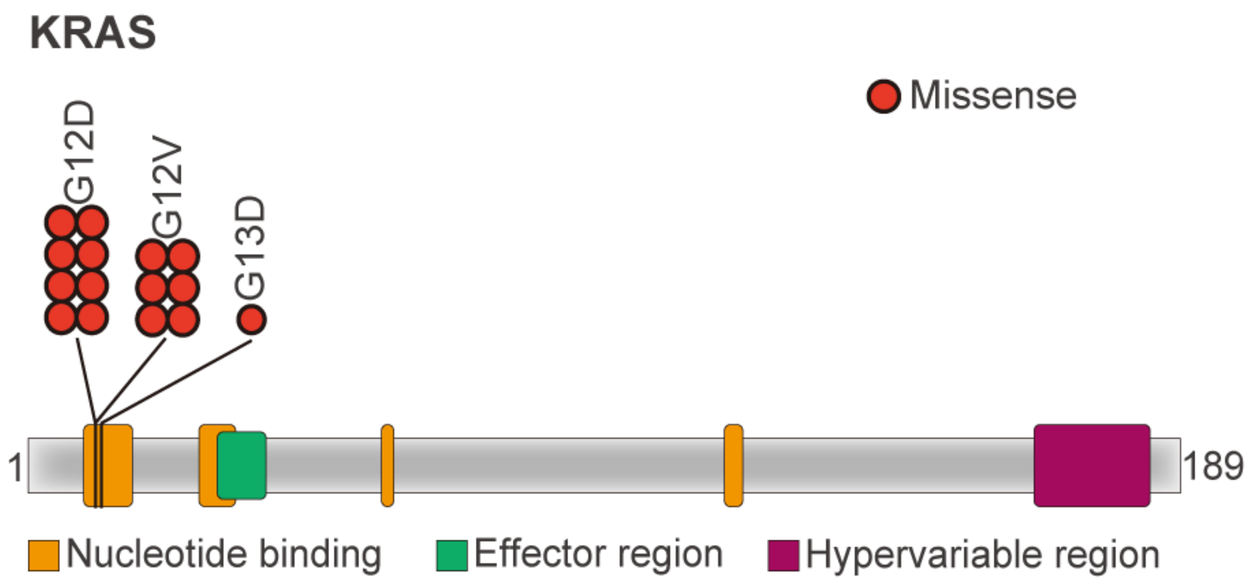

Supplementary Figure 1. Localization of somatic mutations in *KRAS* identified by target-gene sequencing.

A lollipop plot shows the specific locations of the identified somatic mutations in *KRAS* from adenomyosis samples, along with the known domain structures of the protein. Numbers indicate amino acid residues. Each circle corresponds to an independent somatic mutation event.

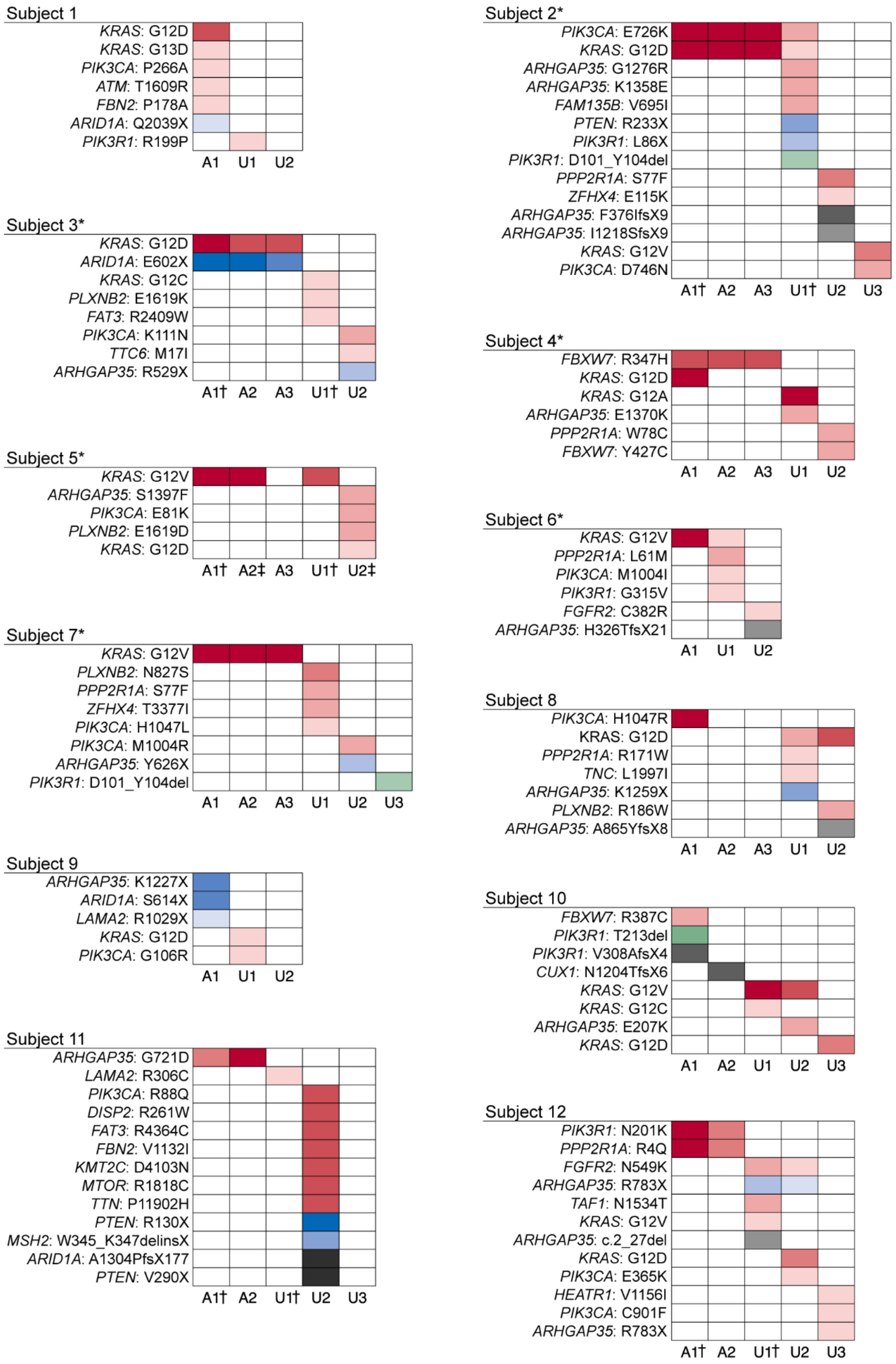
Subject 1
KRAS: G12D
KRAS: G13D
PIK3CA: P266A
ATM: T1609R
FBN2: P178A
ARID1A: Q2039X
PIK3R1: R199P
A1 U1 U2
Subject 2*
PIK3CA: E726K
KRAS: G12D
ARHGAP35: G1276R
ARHGAP35: K1358E
FAM135B: V695I
PTEN: R233X
PIK3R1: L86X
PIK3R1: D101_Y104del
PPP2R1A: S77F
ZFHX4: E115K
ARHGAP35: F376IfsX9
ARHGAP35: I1218SfsX9
KRAS: G12V
PIK3CA: D746N
A1† A2 A3 U1† U2 U3
Subject 3*
KRAS: G12D
ARID1A: E602X
KRAS: G12C
PLXNB2: E1619K
FAT3: R2409W
PIK3CA: K111N
TTC6: M17I
ARHGAP35: R529X
A1† A2 A3 U1† U2
Subject 4*
FBXW7: R347H
KRAS: G12D
KRAS: G12A
ARHGAP35: E1370K
PPP2R1A: W78C
FBXW7: Y427C
A1 A2 A3 U1 U2
Subject 5*
KRAS: G12V
ARHGAP35: S1397F
PIK3CA: E81K
PLXNB2: E1619D
KRAS: G12D
A1† A2‡ A3 U1† U2‡
Subject 6*
KRAS: G12V
PPP2R1A: L61M
PIK3CA: M1004I
PIK3R1: G315V
FGFR2: C382R
ARHGAP35: H326TfsX21
A1 U1 U2
Subject 7*
KRAS: G12V
PLXNB2: N827S
PPP2R1A: S77F
ZFHX4: T3377I
PIK3CA: H1047L
PIK3CA: M1004R
ARHGAP35: Y626X
PIK3R1: D101_Y104del
A1 A2 A3 U1 U2 U3
Subject 8
PIK3CA: H1047R
KRAS: G12D
PPP2R1A: R171W
TNC: L1997I
ARHGAP35: K1259X
PLXNB2: R186W
ARHGAP35: A865YfsX8
A1 A2 A3 U1 U2
Subject 9
ARHGAP35: K1227X
ARID1A: S614X
LAMA2: R1029X
KRAS: G12D
PIK3CA: G106R
A1 U1 U2
Subject 10
FBXW7: R387C
PIK3R1: T213del
PIK3R1: V308AfsX4
CUX1: N1204TfsX6
KRAS: G12V
KRAS: G12C
ARHGAP35: E207K
KRAS: G12D
A1 A2 U1 U2 U3
Subject 11
ARHGAP35: G721D
LAMA2: R306C
PIK3CA: R88Q
DISP2: R261W
FAT3: R4364C
FBN2: V1132I
KMT2C: D4103N
MTOR: R1818C
TTN: P11902H
PTEN: R130X
MSH2: W345_K347delinsX
ARID1A: A1304PfsX177
PTEN: V290X
A1† A2 U1† U2 U3
Subject 12
PIK3R1: N201K
PPP2R1A: R4Q
FGFR2: N549K
ARHGAP35: R783X
TAF1: N1534T
KRAS: G12V
ARHGAP35: c.2_27del
KRAS: G12D
PIK3CA: E365K
HEATR1: V1156I
PIK3CA: C901F
ARHGAP35: R783X
A1† A2 U1† U2 U3

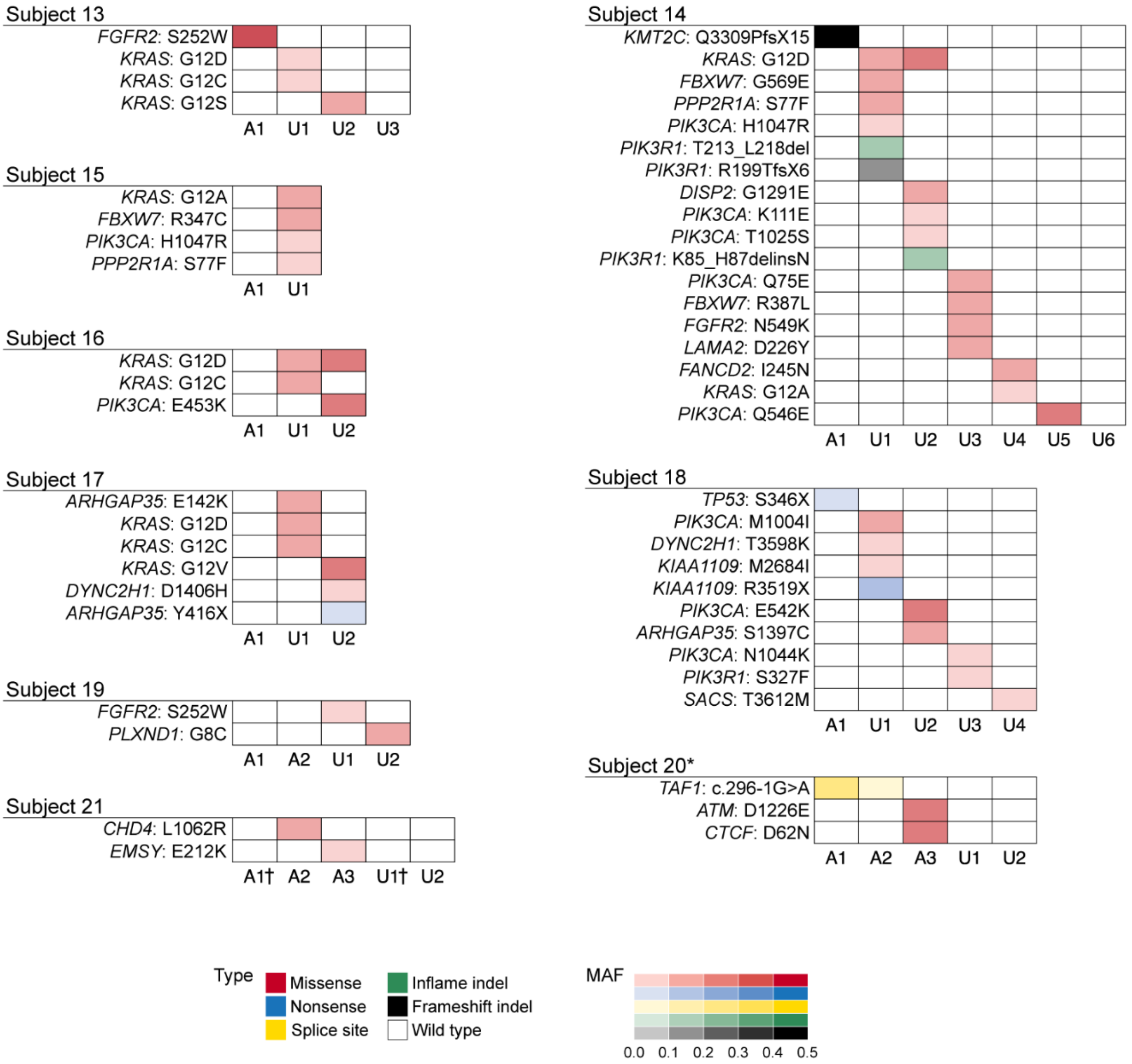

Supplementary Figure 2. Heatmap of mutation profiling detected by target-gene sequencing in all subjects.

Each heatmap shows all somatic mutations detected by target-gene sequencing. Color and its density indicate type and mutant allele frequency, respectively. * indicates subjects who have whole-exome sequencing results. † and ‡ indicate samples taken from the same block for adenomyosis and endometrium.

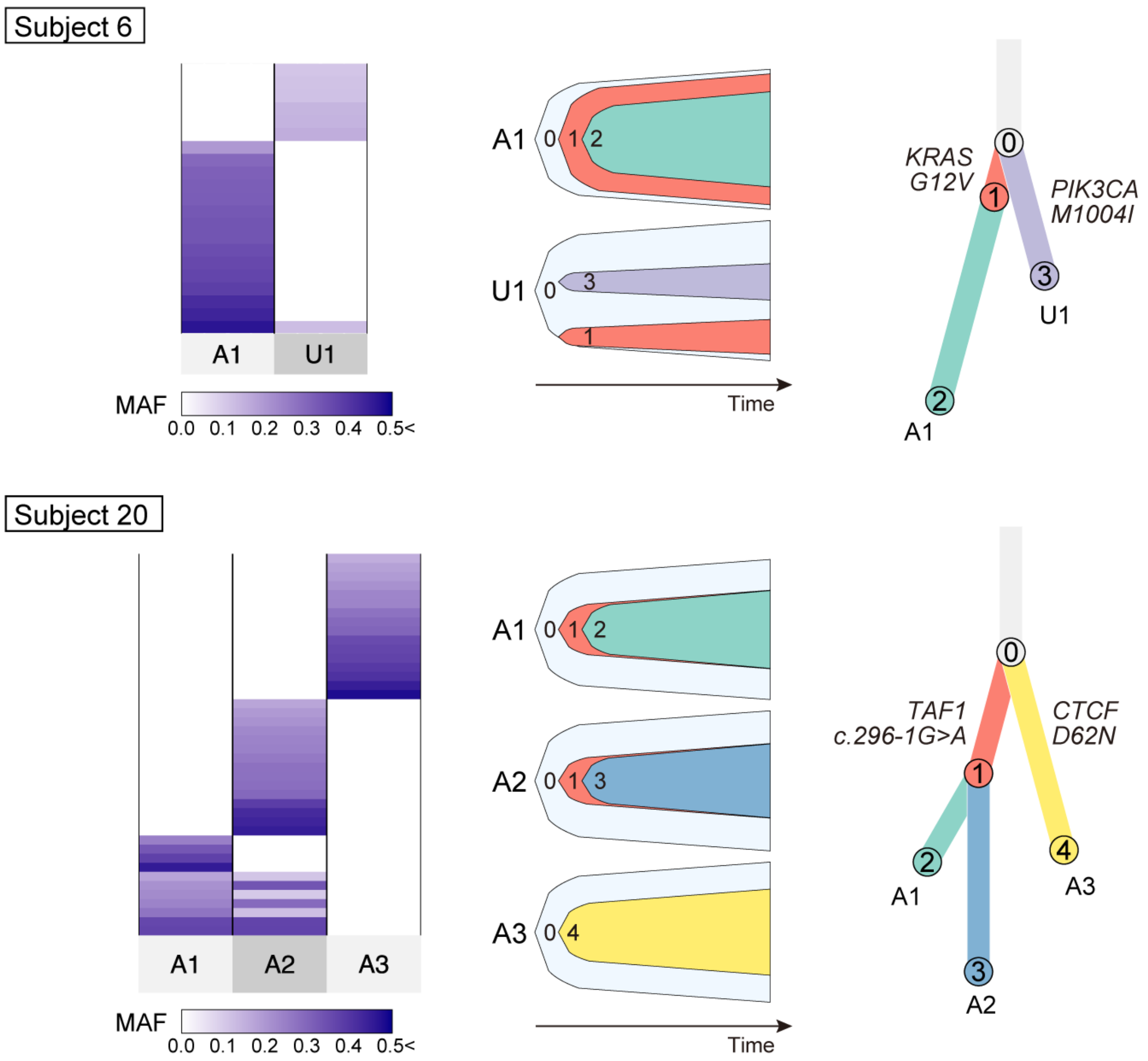

Supplementary Figure 3. Clonal relationships in the remaining subjects with whole-exome sequencing analysis.

The left panel in each subject shows the shared pattern of somatic mutations detected by whole-exome sequencing. Color density indicates the mutant allele frequency of each somatic mutation. The middle to right panel shows fish plots and a branch-based clonal evolution tree, created by clustering with PyClone and ordering with ClonEvol. Fish plots show the process of clonal evolution in each sample. In a clonal evolution tree, mutations in cancer-associated genes were assigned to branches based on the result of mutation clustering by PyClone. The identifiers of samples are indicated beside a node if the clone was observed in the corresponding samples at the time when the sample was taken. The lengths of the branches are associated with the number of somatic mutations. A indicates adenomyosis, and U indicates uterine endometrium.
